# Electronic symptom monitoring after lung cancer surgery: establishing a core set of patient-reported outcomes for surgical oncology care in a longitudinal cohort study

**DOI:** 10.1097/JS9.0000000000001855

**Published:** 2024-06-20

**Authors:** Hongfan Yu, Cheng Lei, Xing Wei, Yaqin Wang, Wei Xu, Li Tang, Wei Dai, Jia Liao, Yang Pu, Ruoyan Gong, Xueyao Su, Qingsong Yu, Jiayuan Zhang, Lijun Zhang, Yanyan Huang, Xiang Zhuang, Jin Bai, Zhibiao Wang, Qiang Li, Qiuling Shi

**Affiliations:** a State Key Laboratory of Ultrasound in Medicine and Engineering, College of Biomedical Engineering, Chongqing Medical University, Chongqing, China; b School of Public Health, Chongqing Medical University, Chongqing, China; c Department of Thoracic Surgery, Sichuan Clinical Research Center for Cancer, Sichuan Cancer Hospital & Institute, Sichuan Cancer Center, Affiliated Cancer Hospital of University of Electronic Science and Technology of China, Chengdu, Sichuan, China; d Shaoxing Second Hospital, Shaoxing, Zhejiang, China; e School of General Education, Chongqing College of Traditional Chinese Medicine, Chongqing, China; f Chengdu Center for Disease Control and Prevention, Chengdu, Sichuan, China

**Keywords:** eHealth, lung surgery, patient-reported outcome measure, surgical practice, symptom management

## Abstract

**Background:**

Electronic symptom monitoring via patient-reported outcomes in surgical oncology is limited owing to lengthy instruments and non-specific items in common patient-reported outcome instruments. To establish electronic symptom monitoring through a clinically relevant and fit-for-purpose core set of patient-reported outcome in patients undergoing lung cancer surgery.

**Materials and methods:**

One qualitative (Cohort 1) and two prospective studies (Cohorts 2 and 3) were conducted between 2018 and 2022. Patients undergoing lung cancer surgery were recruited. Items of symptoms and daily functioning were generated through extensive interviews in Cohort 1 and incorporated into a smartphone-based platform to establish the electronic Perioperative Symptom Assessment for Lung surgery (ePSA-Lung). This instrument was finalized and validated in Cohort 2. Patients in Cohort 3 were longitudinally monitored for the first-year post-surgery using the validated ePSA-Lung.

**Results:**

In total, 1037 patients scheduled for lung cancer surgery were recruited. The 11-item draft PSA-Lung was generated based on qualitative interview with 39 patients and input from a Delphi study involving 42 experts. A 9-item ePSA-Lung was finalized by assessing 223 patients in the validation cohort; the results supported the instrument’s understandability, reliability, sensitivity, and surgical specificity. In Cohort 3 (*n*=775), compliance ranged from 63.21 to 84.76% during the 1-year follow-up after discharge. Coughing, shortness of breath, and disturbed sleep were the most severe symptoms after discharge. Longitudinally, patients who underwent single-port video-assisted thoracic surgery had a lower symptom burden than those who underwent multi-port video-assisted thoracic surgery or thoracotomy (all symptoms, *P*<0.001).

**Conclusions:**

The ePSA-Lung is valid, concise, and clinically applicable as it supports electronic symptom monitoring in surgical oncology care. The need for long-term extensive care was identified for patients after discharge, even in early-stage cancer with potential curative treatment.

## Introduction

Highlights

- Electronic symptom monitoring in lung surgical oncology care.
- Electronic Perioperative Symptom Assessment for Lung surgery (ePSA-Lung) is valid, concise, and clinically applicable instrument.
- Developing an electronic patient-reported outcome (ePRO) instrument to be implemented in perioperative care.
- Profiling short- and long-term symptom burden 1-year post lung cancer surgery.

Enhanced recovery after surgery and minimally invasive techniques have reduced hospital stays after lung cancer surgery^1^. However, substantial symptoms significantly delay patients’ return to normal life^2^ and the initiation of adjuvant therapies^3^. Randomized clinical trials have demonstrated that digital techniques, such as smartphones, web-based or interactive voice response systems, and patient-reported outcome (PRO)-based symptom management, enhance recovery, improve daily functioning and quality-of-life^4,5^, optimize healthcare utilization^6^, and prolong survival^7–9^. Electronic symptom reporting, triggering immediate responses when thresholds are surpassed during scheduled assessments, is an eagerly anticipated practice in postoperative care. Hence, it is under study for routine implementation in surgical settings^6–8^.

To date, electronic PRO (ePRO) has primarily benefited metastatic cancer patients^3^. A few studies have validated its impact on patients undergoing cancer surgery in clinical practice. However, in these studies, the patients and providers were burdened with lengthy^10^, multi-domain instruments^11^, with low compliance (58.1% by week 18)^12^. Moreover, these internationally validated PRO instruments^13–17^ were mainly developed for patients undergoing systemic therapy and lacked items relevant to perioperative care. Dynamic postoperative rehabilitation of surgical patients requires frequent assessments. Best practices for digital monitoring recommend a simple questionnaire of 10–15 actionable symptoms^3^. Therefore, continuing efforts to condense PRO items may enhance their usability for routine symptom monitoring^18–20^.

In this prospective observational study, we aimed to develop a concise core set of PRO for frequent and sensitive symptom monitoring after lung cancer surgery, named the electronic Perioperative Symptom Assessment for Lung surgery (ePSA-Lung). Following the United States Food and Drug Administration (FDA) guidances^19–22^, we hypothesized that the ePSA-Lung would be: (1) psychometrically valid for measuring perioperative symptoms; (2) sensitive and responsive to surgical insults, clinical outcomes, and recovery status; and (3) easily built into an ePRO monitoring system^2^ to profile short- and long-term symptom burden during the first-year after lung cancer surgery, using daily, weekly, and monthly assessment schedules. Ultimately, our goal was to develop an ePRO that can be implemented in perioperative care, utilizing a brief and fit-for-purpose set of PRO.

## Methods

### Study design and ethical approval

The Ethics Committee for Medical Research and New Medical Technology of Sichuan Cancer Hospital approved this prospective observational study (CN-PRO-Lung 3), which was registered with the Chinese Clinical Trials Registry. This work has been reported in line with the STROCSS criteria (Supplemental Digital Content 1, http://links.lww.com/JS9/C815)^23^. All participants provided written or electronic informed consent.

### Participants

Following the FDA guidelines, we summarized the process for item generation, cognitive debriefing, psychometric validation, and clinical application (Supplemental Table 1, Supplemental Digital Content 2, http://links.lww.com/JS9/C816)^19–22^. This study comprised three cohorts of patients undergoing lung cancer surgery. The item pool was generated from qualitative interviews in Cohort 1 with the information saturation of sample size, and the ePSA-Lung was drafted after consulting an expert panel. The core set of items was finalized through psychometric validation in Cohort 2 for the 10 times in items number of sample size, while Cohort 3 was used to demonstrate its applicability in routine symptom monitoring during the first-year post-discharge. Patients ≥18 years old who spoke Chinese and understood the study’s content were recruited from November 2018 to May 2022, with exclusions for cognitive impairments, or ineffective communication. Patients who did not receiving surgery treatment would be excluded during data analysis.

### ePSA-Lung system

We integrated the draft PSA-Lung generated from Cohort 1 into an established and previously validated ePRO symptom monitoring system^2^. Patients provided data through a WeChat mini-program on their smartphones, either at home or in the hospital. Nonreporting triggered smartphone reminders at 7 pm, followed by a phone call if patients had not reported within three scheduled assessments. Risk control alerts, triggered by pain or distress above the pre-set thresholds (≥7), were sent to the clinician team. Following symptom control guidelines^24,25^, clinicians contacted patients for further actions.

### Cohort 1: Item generation

We generated a candidate item pool by inviting patients with primary lung cancer to qualitative interviews before discharge (Supplemental Method, Supplemental Digital Content 2, http://links.lww.com/JS9/C816)^26^. The experts evaluated each item’s clinical relevance through two rounds of evaluation, rating from 0 (not relevant) to 10 (very relevant). Items with an average score of greater than or equal to 5 and variation of less than or equal to 2 points across two rounds were retained in the provisional PSA-Lung.

### Cohort 2: Cognitive debriefing and psychometric validation

The draft PSA-Lung was integrated into an established ePRO platform to create the ePSA-Lung. Patients scheduled for surgery in Cohort 2 completed the ePSA-Lung 1–3 days before-surgery and daily after surgery until discharge. They also completed the MD Anderson Symptom Inventory for Lung Cancer (MDASI-LC) at baseline and on the second postoperative day (POD2). Cognitive debriefing assessed item comprehension, patient comfort, and acceptability after the initial ePSA-Lung completion^21^.

### Item finalization

Items were selected using five different indicators to meet the required standard criteria, ultimately generating the final ePSA-Lung version (Supplemental Method, Supplemental Digital Content 2, http://links.lww.com/JS9/C816)^27–29^.

### Reliability and validity

Cronbach’s α for internal consistency and the intraclass correlation coefficient (ICC) for test-retest reliability were evaluated for the final ePSA-Lung (Supplemental Method, Supplemental Digital Content 2, http://links.lww.com/JS9/C816)^30^. A value of 0.7 or higher for Cronbach’s α and ICC indicated good reliability. Criterion validity was determined by calculating Pearson’s correlation coefficients between each ePSA-Lung item and the corresponding item in the MDASI-LC.

### Sensitivity and responsiveness

We evaluated known-group validity by differentiating patients with varying statuses or outcomes. Cohen’s *d* effect sizes and independent sample t-tests were used to compare symptom scores across surgical approaches [single-port video-assisted thoracic surgery (VATS) vs. other approaches (multi-port VATS or thoracotomy)], lung disease types (lung cancer vs. benign tumors), the number of thoracic drainage tubes (≤1 vs. 2), and postoperative complications (yes vs. no). We evaluated item responsiveness by comparing baseline and discharge scores using paired *t*-tests. Linear mixed effect (LME) models were employed to assess the sensitivity and responsiveness by examining symptom differences over time, between two surgical approaches, hypothesized to have varying levels of impact on patients’ well-being differently.

### Cohort 3: Clinical application

Patients in Cohort 3, scheduled for lung surgery, were prospectively enrolled and retained upon pathological confirmation of lung cancer. The ePSA-Lung assessments were administered preoperatively, daily during hospitalization and up to 1-month post-discharge, weekly during the second and third months after discharge, and monthly for the remainder of the first year.

### Statistical analyses

We evaluated ePSA-Lung acceptance through patient compliance (Supplemental Method, Supplemental Digital Content 2, http://links.lww.com/JS9/C816) and response burden (completion times). The top five symptoms were those with the highest rate of moderate-to-severe levels (scored ≥4) throughout the 1-year follow-up. We used LME models to examine differences in symptom trajectories between single-port VATS and other approaches during hospitalization, adjusting for age, sex, education level, smoking status, Charlson Comorbidity Index score, number of chest tubes, and the pathology tumor node metastasis stage. Piece-wise LME models were fitted to describe symptom trajectories between surgical approaches over time, segmented into five periods: (1) baseline to POD1; (2) POD1 to POD4; (3) the first month after discharge; (4) months 2–3 after discharge; and 5) months 4–12 after discharge. Baseline variables that differed between groups were controlled in all models. A two-tailed *P* value less than 0.05 was considered statistically significant, and analysis was performed using SAS version 9.4.

## Results

### Patient characteristics

We recruited 39 patients with primary lung cancer for the qualitative interviews (Cohort 1), 223 for psychometric validation (Cohort 2), and 775 for the clinical application (Cohort 3), with average ages of 59.50±8.32, 53.90±10.71, and 52.74±11.14 years. More than half of patients underwent single-port VATS, over 95% had a good performance status preoperatively, and with 10.3–11.4% incidences in-hospital complications druing cohorts 2 and 3 (Table 1).

**Table 1:** Demographic and clinical characteristics.

Characteristics	Cohort 1 (*n*=39)	Cohort 2 (*n*=223)	Cohort 3 (*n*=775)
Age, mean ± SD, years	59.50±8.32	53.90±10.71	52.74±11.14
<55, *n* (%)	10 (25.6)	113 (50.7)	422 (54.4)
≥55, *n* (%)	29 (74.4)	110 (49.3)	353 (45.6)
BMI, mean ± SD, kg/m^2^	23.03±3.11	22.94±2.96	22.81±3.08
Sex, *n* (%)			
Male	24 (61.5)	93 (41.7)	273 (35.2)
Female	15 (38.5)	130 (58.3)	502 (64.8)
Education level, *n* (%)			
Senior school graduate or below	20 (51.3)	119 (53.4)	436 (56.3)
Above senior school graduate	16 (41.0)	104 (46.6)	339 (43.7)
Unknown	3 (7.7)	0 (0)	0
Smoking status, *n* (%)			
Never smoke	21 (53.8)	158 (70.9)	621 (80.1)
Current smoker	13 (33.3)	48 (21.5)	105 (13.6)
Former smoker	5 (12.8)	17 (7.6)	49 (6.3)
CCI score, *n* (%)			
≤1	19 (48.7)	145 (65.0)	525 (67.7)
>1	20 (51.3)	78 (35.0)	250 (32.3)
ECOG-PS, *n* (%)			
0	NA	217 (97.3)	768 (99.1)
≥1	NA	6 (2.7)	7 (0.9)
Surgical approach, *n* (%)			
Single-port VATS	21 (53.9)	119 (53.4)	485 (62.6)
Two-port VATS	5 (12.8)	13 (5.8)	67 (8.7)
Three-port VATS	6 (15.4)	41 (18.4)	73 (9.4)
Four-port VATS	2 (5.1)	30 (13.5)	17 (2.2)
Thoracotomy	3 (7.7)	13 (5.8)	16 (2.1)
VATS conversion to thoracotomy	2 (5.1)	7 (3.1)	27 (3.5)
RATS	0	0	90 (11.6)
The number of thoracic drainage tubes, *n* (%)			
0	0	2 (0.9)	0
1	19 (48.7)	102 (45.7)	644 (83.1)
2	20 (51.3)	119 (53.4)	131 (16.9)
pTNM stage (8th Edition), *n* (%)			
≤Ⅰ	25 (64.1)	165 (74.0)	704 (90.8)
>Ⅰ	14 (35.9)	24 (10.7)	71 (9.2)
Not applicable (lung benign tumor)	NA	34 (15.3)	NA
PHS, median (IQR), days	7 (5–9)	4 (4–6)	4 (3–5)
In-hospital complications, *n* (%)			
Yes	16 (41.0)	23 (10.3)	88 (11.4)
No	23 (56.0)	200 (89.7)	687 (88.6)
Time to complete ePSA-Lung, median (IQR), sec			
Pre-operation	NA	NA	NA
Post-operation	NA	91 (57–176)	96 (61–154)
Post-discharge	NA	NA	39 (26–58)

CCI, Charlson Comorbidity Index; ECOG-PS, Eastern Cooperative Oncology Group-Performance Status; ePSA-Lung, electronic Perioperative Symptom Assessment for Lung surgery; IQR, interquartile range; NA, not available; PHS, postoperative length of hospital stays; pTNM, pathology tumor node metastasis; RATS, robotic-assisted thoracoscopic surgery; VATS, video-assisted thoracic surgery.

### Cohort 1: Item generation

On the 18-item list (Supplemental Table 2, Supplemental Digital Content 2, http://links.lww.com/JS9/C816) generated by the qualitative interview^26^, a panel comprising 42 experts [37 clinicians, 4 nurses, and 1 researcher; 64.3% (27/42) with over 10 years in thoracic surgery] identified 11 items, including pain, cough, shortness of breath, hoarseness, disturbed sleep, fatigue, drowsiness, anxiety, distress, walking difficulty, and activity limitation. The instrument employed a 24-hour recall period and an 11-point scale ranging from 0 (not present) to 10 (as bad as imaginable).

### Cohort 2: Item finalization and measurement characterization

#### Cognitive debriefing

Overall, 218 patients underwent cognitive debriefing following the ePSA-Lung assessment. Of these, 209 (95.8%) reported that the instrument was easy to complete, 201 (92.2%) deemed it easy to understand, and 209 (95.8%) stated that the 0–10 numeric scale was easy to understand. No other symptoms were suggested in response to an open question posed to all patients.

#### Item finalization

We collected data from 223 hospitalized patients (Supplemental Table 3, Supplemental Digital Content 2, http://links.lww.com/JS9/C816). The items that exhibited a floor effect above the threshold were “hoarseness” (37.1%), “distress” (34.5%), and “anxiety” (33.7%). The expert panel suggested removing “hoarseness” and retaining only one item between “distress” and “anxiety” due to their strong correlation (r=0.76, *P*<0.001). When compared to “distress,” “anxiety” showed a lower average score (2.16 vs. 2.41), and patients experienced greater difficulty in understanding the concept of “anxiety” during cognitive debriefing. Consequently, it was decided to remove both “hoarseness” and “anxiety,” leaving 9 items for further validation as the final ePSA-Lung.

#### Reliability and validity

The ePSA-Lung showed excellent internal consistency (Cronbach’s ɑ=0.91) and test-retest reliability (ICCs, 0.86–0.95). The correlation coefficients between items from ePSA-Lung and their counterparts in MDASI-LC ranging from 0.72 to 0.84, except for “distress” (r=0.62). Compared with MDASI-LC, ePSA-Lung presented slightly higher patient compliance in baseline (100% vs. 98.2%, *P*=0.12) and POD2 (90.09% vs. 86.93%, *P*=0.30).

#### Sensitivity and responsiveness

**Figure 1:**
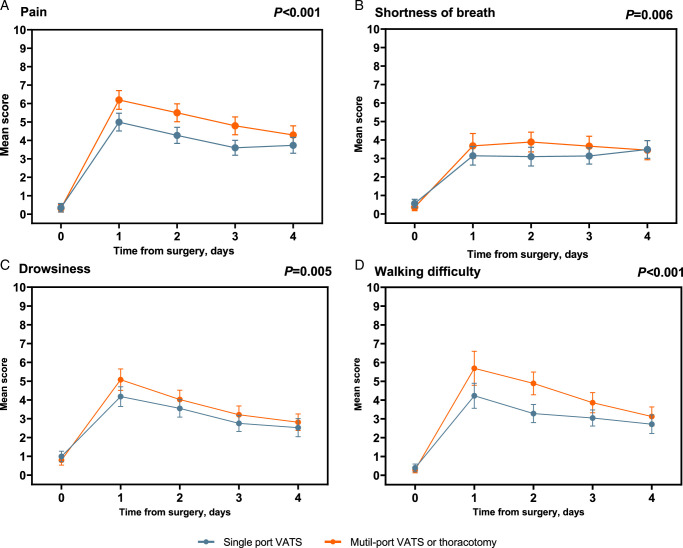
Mean scores of electronic Perioperative Symptom Assessment for Lung surgery (ePSA-Lung) from baseline to postoperative day 4, by surgical approaches (Cohort 2). The points represent actual mean value at each time point and the vertical bars represent its 95% CI. Mean scores of items on the electronic Perioperative Symptom Assessment for Lung surgery (ePSA-Lung) instrument: (A) Pain scores. (B) Shortness of breath scores. (C) Drowsiness scores. (D) Walking difficulty scores. *P* values for the difference between single-port VATS and multi-port VATS groups from baseline to the 4th postoperative day were obtained from linear mixed effect models, adjusted for smoking status, number of chest tube, and the pathology tumor node metastasis stage. In these models, the dependent variable was the score of each item, considered as a continuous variable, and the independent variables were time (also as a continuous variable), the surgical approach groups, the interaction between time and the surgical approach groups, and previously mentioned adjusted variables. The fixed effects of all independent variables and random effects for intercept and time were estimated using the maximum likelihood estimation method. The covariance structure was specified as the first-order autoregressive. VATS, Video-Assisted Thoracic Surgery.

Patients with lung cancer significantly scored higher in certain symptoms than those with benign disease, those with single-port VATS scored lower than those with other approaches, those with two chest tubes scored higher than those with one, and those with postoperative complications scored higher than those without (Supplemental Table 4 and Table 5, Supplemental Digital Content 2, http://links.lww.com/JS9/C816). Each ePSA-Lung item significantly differed between baseline and discharge day (Supplemental Table 6, Supplemental Digital Content 2, http://links.lww.com/JS9/C816), displaying large effect sizes (Cohen’s *d* >0.7, except 0.34 for “fatigue” and 0.2 for “walking difficulty”). Moreover, single-port VATS resulted in significantly lower “pain,” “shortness of breath,” “drowsiness,” and “walking difficulty” (*P*<0.01) compared with other approaches (Fig. 1; Supplemental Figure 1, Supplemental Digital Content 2, http://links.lww.com/JS9/C816). Longitudinally, symptoms were significantly decreased during POD1 to POD4 (*P*<0.001).

### Cohort 3: Clinical applicability

#### Compliance and response burden

**Figure 2:**
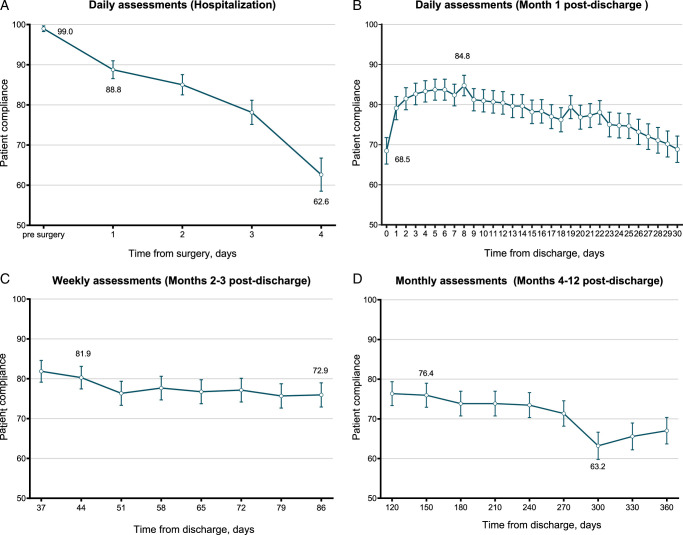
Patient compliance from baseline to the end of the first year after discharge (Cohort 3). The points represent the actual proportion of patient compliance rate at each time point and the vertical bars represent the 95% CI. The patient compliance rates of the electronic Perioperative Symptom Assessment for Lung surgery (ePSA-Lung) during hospitalization and within the 1-year follow-up after discharge. The patient compliance rate was calculated by dividing the number of patients who successfully completed the ePSA-Lung by the number of patients required to complete it at pre-determined time points. This was done under four different schedules: (1) daily assessments while in the hospital (A); (2) daily assessments during the first month post-discharge (B); (3) weekly assessments during the second month to the third month post-discharge (C); and (4) monthly assessments from the fourth to the twelfth month post-discharge (D).

Patients took a median of 96 s to complete the ePSA-Lung during hospitalization and 39 s after discharge. Patient compliance was 99% before-surgery, varied between 62.6% and 88.8% during hospitalization. Post-discharge compliance ranged from 68.5 to 84.8% for daily assessments, from 72.9 to 81.9% for weekly assessments, and from 63.2 to 76.4% for monthly assessments (Fig. 2; Supplemental Table 7, Supplemental Digital Content 2, http://links.lww.com/JS9/C816). The highest rate of item missing, which was 2.0% for “distress,” occurred on POD2 during hospitalization (Supplemental Table 8, Supplemental Digital Content 2, http://links.lww.com/JS9/C816).

#### Symptom severity

**Figure 3:**
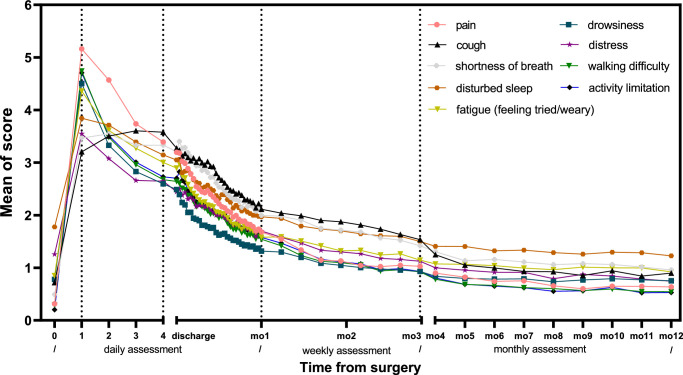
Trajectories of symptoms and physical functioning over the first year of lung cancer surgery (Cohort 3). The trajectory on the mean score of each item on the electronic Perioperative Symptom Assessment for Lung surgery from baseline to the end of the first year after discharge. The entire follow-up period was divided into five segments: (1) from baseline to the first postoperative day (POD1); (2) from POD1 to POD4; (3) the first month following discharge; (4) from the second to the third month post-discharge; and (5) from the fourth to the twelfth month post-discharge.

The mean trajectory of each item following surgery is depicted in Figure 3. The top five moderate-to-severe symptoms during hospitalization were “pain” (43.8%), “disturbed sleep” (40.0%), “fatigue” (37.8%), “cough” (35.4%), and “shortness of breath” (35.3%). While after discharge, the top five symptoms were “cough” (22.1%), “shortness of breath” (21.5%), “disturbed sleep” (21.3%), “fatigue” (15.2%), and “pain” (15.2%). Using a severe symptom threshold (≥7), we found that 70.5% of patients reported one or more severe symptoms after operation. After discharge, 41.68% reported at least one severe symptom during the first month post-discharge, 12.3% during months 2–3, and 9.9% at the end of the first year.

#### Symptoms changing over time

On average, each item score significantly increased from pre-surgery to POD1, then decreased from POD1 to month 12 after discharge (all *P*<0.05, Table 2). Among all nine items, “pain” showed the sharpest increase on POD1, jumping by 5.48 points (95% CI, 5.27–5.68), whereas “disturbed sleep” exhibited the smallest increase of 2.35 (95% CI, 2.1–2.6). During POD1 to POD4, there was a slight increase (0.16) in “cough”, while the other items decreased by 0.08–0.61 points.

**Table 2:** Changes of ePSA-Lung scores over 1 year (Cohort 3).

Items of ePSA-Lung	Estimate (95% CI) of ePSA-lung item scores changing over time^a^				
	Baseline to POD1	POD1 to POD4(daily)	Discharge days 1–30 (daily)	Discharge months 2–3 (weekly)	Discharge months 4–12 (monthly)
Pain^a^	5.48 (5.27–5.68)^b^	−0.61 (−0.69 to −0.53)^b^	−0.006 (−0.009 to −0.003)^b^	−0.06 (−0.07 to −0.05)^b^	−0.11 (−0.12 to −0.09)^b^
Cough	2.35 (2.15–2.55)^b^	0.16 (0.08 to −0.23)^b^	−0.005 (−0.008 to −0.001)^c^	−0.05 (−0.06 to −0.04)^b^	−0.13 (−0.14 to −0.12)^b^
Shortness of breath	3.08 (2.88–3.29)^b^	−0.08 (−0.16 to 0)^c^	−0.008 (−0.011 to −0.005)^b^	−0.06 (−0.07 to −0.05)^b^	−0.11 (−0.12 to −0.10)^b^
Disturbed sleep	2.35 (2.1–2.6)^b^	−0.23 (−0.33 to −0.13)^b^	−0.006 (−0.009 to −0.002)^c^	−0.04 (−0.06 to −0.03)^b^	−0.08 (−0.09 to −0.06)^b^
Fatigue (feeling tired/weary)	3.97 (3.76–4.19)^b^	−0.49 (−0.58 to −0.4)^b^	−0.009 (−0.012 to −0.006)^b^	−0.05 (−0.06 to −0.04)^b^	−0.08 (−0.10 to −0.07)^b^
Drowsiness	4.35 (4.11–4.58)^b^	−0.70 (−0.79 to −0.61)^b^	−0.007 (−0.009 to −0.004)^b^	−0.04 (−0.05 to −0.03)^b^	−0.07 (−0.08 to −0.06)^b^
Distress	2.67 (2.44–2.89)^b^	−0.36 (−0.46 to −0.27)^b^	−0.002 (−0.005 to 0)	−0.04 (−0.05 to −0.03)^b^	−0.08 (−0.09 to −0.07)^b^
Walking difficulty	5.10 (4.87–5.34)^b^	−0.75 (−0.84 to −0.67)^b^	−0.005 (−0.007 to −0.002)^b^	−0.05 (−0.06 to −0.03)^b^	−0.09 (−0.10 to −0.08)^b^
Activity limitation	5.14 (4.91–5.37)^b^	−0.73 (−0.81 to −0.64)^b^	−0.006 (−0.008 to −0.003)^b^	−0.06 (−0.07 to −0.05)^b^	−0.10 (−0.11 to −0.09)^b^

ePSA-Lung, electronic Perioperative Symptom Assessment for Lung surgery; POD, postoperative day.

a All estimates of item scores changing over time were obtained from piece-wise linear mixed effect models, adjusted for age, sex, education level, smoking status, Charlson Comorbidity Index score, number of chest tubes, and the pathology tumor node metastasis (pTNM) stage. The time period was divided into five phases: (1) baseline to POD1; (2) POD1 to POD4; (3) the first month after discharge; (4) months 2–3 after discharge; and (5) months 4–12 after discharge.

b *P*<0.001;

c *P*<0.05.

The trajectory of symptoms stratified by surgical approaches is depicted (Supplemental Figure 2, Supplemental Digital Content 2, http://links.lww.com/JS9/C816). Compared to patients undergoing multi-port VATS or thoracotomy, those receiving single-port VATS showed significantly lower severity in longitudinal trajectories of “pain,” “walking difficulty,” “distress,” “activity limitation,” “shortness of breath,” and “disturbed sleep” during hospitalization post-surgery. Moreover, in the first-year post-discharge, those patients consistently reported lower severity levels across all 9 items of ePSA-Lung.

## Discussion

We developed and validated the ePSA-Lung, a concise instrument designed to frequently monitor symptoms via smartphones after lung cancer surgery. Using the ePSA-Lung, we were able to profile symptom relief and functional recovery during the first-year post-discharge. We demonstrated that patients undergoing single-port VATS experienced a lower symptom burden and better functional status compared to those receiving multi-port VATS or thoracotomy. We anticipate this sensitive and responsive symptom set will improve ePRO utilization in perioperative practices and researches, due to its capability to track recovery status and differentiate patients’ responses to surgical insults.

A core set of PRO items is crucial for enhancing their clinical application in patient care^31^. However, the item list should be minimally burdensome and understandable since frequent measurements are necessary, due to significant postoperative symptom changes. Thus, we developed a 9-item symptom assessment that can be completed in merely a minute with a 24-h recall period. The most reported symptoms (pain, fatigue, cough, disturbed sleep, and shortness of breath) in our study were perceived by both healthcare providers and patients, consistent with previous studies^2,32,33^. Following prior recommendations^19–21^, we incorporated two physical functioning items, activity limitation, and walking difficulty. These validated items reflected the surgical impact on daily living^34^, and are applicable even when patients cannot complete a performance-based assessment, like the Timed Up & Go test^35^. Given that cognitive debriefing identified no additional concerns, our study inferred that the ePSA-Lung, despite being shorter than other instruments, adequately captures the patient’s perioperative experience.

While most core sets of PROs are produced using qualitative approaches, their reliability, validity, and responsiveness are challenged^36^. Therefore, after compiling a list of nine items, we established measurement properties in patients with lung cancer surgery, adhering to FDA recommendations. The known-group validity demonstrated that ePSA-Lung can differentiate patient status and evaluate less insulting treatment approaches^34^. This discriminative property enables the use of PROs as clinical endpoints in randomized controlled trials and observational studies, facilitating the quantification of treatment superiority^37–39^.

The primary recovery concerns for both patients and clinicians are symptom relief and functional recovery. This necessitates the use of an instrument that can respond to changes over time^19–21^. The top five symptoms identified in our study are consistent with those measured by the full-length MDASI-LC^2,26,40–44^. Additionally, we quantified symptom trajectories up to one year after surgery, subdividing this period into five clinically pertinent segments: pre-surgery, post-surgery hospitalization, and one month, two to three months, and three to twelve months post-discharge. This segmented analysis affirmed the responsiveness of the ePSA-Lung throughout the full course of recovery and provides reference values for future studies and patient care plans regarding sample size calculation, budget estimation, and resource allocation.

The longitudinal cohort design facilitated the evaluation of ePSA-Lung in various perioperative phases using appropriate assessment schedules, including daily assessment in the early postoperative phase (during hospitalization and 1-month post-discharge), and transitioning to less frequent, weekly or monthly assessments in subsequent phases. Given the concise format and easily understandable 0–10 scale, the assessment time was reduced to 1–2 min, allowing for long-term and daily monitoring of patient recovery trajectories, a feature absent in most previous studies^45–47^. Nevertheless, a frequent assessment schedule might result in less than 50% compliance during longitudinal PRO data collection^48^. In this study, the 9-item instrument yielded a less than 40% missing rate at any time point for all three schedules (daily, weekly, and monthly), which is comparable to the most comprehensive study to date on a similar patient cohort in Canada^44^. Our results suggest the significance and acceptability of the instrument itself^49^, providing a potential solution for adherence issues when implementing ePRO into oncological practice.

These quantified performance characteristics observed throughout the entire recovery course, particularly in patients at home, suggest that the core set of PROs could be effectively utilized in both clinical research and practice. The use of an ePRO monitoring-alerting-intervention system in surgical practice has proven to relieve symptom burden and decrease postoperative complications in randomized clinical trials^2,50,51^. However, implementing it in real-world practice has been challenging, partially due to the burden of frequent PRO monitoring. A concise PRO measure could be promptly transferred to an electronic health record system, improving symptom management, resolving technical issues, and enhancing patient satisfaction.

This study presents a scale derived from an Asian population, considering the cultural adaptability of PROs. The final 9-item PRO aligns with those developed in Western countries, suggesting a cross-cultural consistency in measuring symptoms and physical functioning. Given the high volume of surgeries conducted worldwide, there are extensive practical applications of this set of PROs for surgical practice and research, such as symptom trajectory analysis, monitoring-alerting-intervention models, and routine symptom management^2,10^. Consequently, we anticipate that the ePSA-Lung will offer a wider range of methodological options for related studies, thereby fostering further research.

### Limitations

We recruited patients from a single cancer hospital in China, which may influence the study’s global generalizability. Despite the large sample size illustrated patient heterogeneity, external validation of this instrument in other regions and ethnicities is our further work. Moreover, psychometric validity and clinical application data were only collected electronically during hospitalization and discharge, potentially limiting sample representativeness. Considering the influence of age and education on the utilization of ePRO^48^, we implemented various strategies to improve the accessibility of ePSA-Lung. These strategies included providing timely technical support, designing a simplified yet user-friendly interface, and offering patient education^6,10^. As a result, the demographic characteristics of our cohort closely resembled to those of a nationwide prospective cohort study that included 439 393 participants, indicating the representativeness of our results^52^. Finally, this core set focused solely on symptoms and physical functional impairment, aiming at frequently monitoring patient’s recovery status. A broader list of items (e.g. quality of life) encompassing additional domains might be necessary for less frequent assessment schedules^53^.

## Conclusions

We developed a concise and clinically pertinent ePRO instrument to assess symptom severity and physical functioning in lung cancer surgery. Its quantified psychometric and clinimetric properties make it suitable for electronic evaluation of treatment outcomes, post-surgery monitoring, and enhancement of surgical oncology care.

## Ethical approval

The Ethics Committee for Medical Research and New Medical Technology of Sichuan Cancer Hospital (No. SCCHEC-02-2018-043; November 02, 2018) approved this prospective observational study (CN-PRO-Lung 3).

## Consent

All participants provided written or electronic informed consent.

## Source of funding

This work was supported by the National Key Research and Development Plan for Intergovernmental Cooperation, the Ministry of Science and Technology of China (2022YFE0133100), Chongqing Graduate Student Research Innovation Project (CYB23221), and Beijing Xisike Clinical Oncology Research Foundation Navigator Cancer Research Fund Project (Y-2019AZMS-0486). The funder did not participate in any aspect of the study, including the design, data collection, analysis, and decision to publish.

## Author contribution

All authors took part in the study’s concept/design, data collection, data analysis/interpretation; writing, reviewing or revising the manuscript; approved the final version for submission, and responsibility for accuracy and integrity of all aspects of research. H.Y. and Q.S. had full access to all the data in the study and take responsibility for the integrity of the data and the accuracy of the data analysis. Data analysis: H.Y., C.L., Y.P., W.X., L.T., Q.S.; Drafting of the manuscript: H.Y.; Reviewing the manuscript critically for important intellectual content: All authors; Obtaining funding: H.Y. and Q.S.

## Conflicts of interest disclosure

The authors declare no conflicts of interest.

## Research registration unique identifying number (UIN)

This study was registered with the Chinese Clinical Trials Registry (ChiCTR2000033016; May 18, 2020).

## Guarantor

Qiuling Shi.

## Data availability statement

Please contact the corresponding authors directly. The funder, investigators, and collaborators will review and approve the proposals based on scientific merit and submit them to Ethics Committee for Medical Research and New Medical Technology of Sichuan Cancer Hospital. After approval of a proposal, data can be shared along with anonymized participant data accompanied by a data dictionary through a secure online platform after signing a data access and confidentiality agreement. Data will be available for up to 1 year after a request has been received and approved.

## Provenance and peer review

Not commissioned, externally peer-reviewed.

## Supplementary Material

SUPPLEMENTARY MATERIAL
