# Supplementary material for: Electronic symptom monitoring after lung cancer surgery: establishing a core set of patient-reported outcomes for surgical oncology care in a longitudinal cohort study
Source: Int J Surg. 2024 Jun 20;110(10):6591–600. doi: 10.1097/JS9.0000000000001855 (PMC11486944; doi:10.1097/JS9.0000000000001855)
Supplement: SUPPLEMENTARY MATERIAL [file js9-110-6591-s002.docx]

**Supplementary Online Content**

**eMethods.**

**eFigure 1.** Other symptom trajectories by surgical approaches (Cohort 2)

**eFigure 2.** Symptom trajectories by surgical approaches (Cohort 3)

**eTable 1.** Requirements and methods for the development of PRO instruments

**eTable 2.** Symptoms identified through patient interview and expert evaluation (Cohort 1)

**eTable 3.** Item scores during hospitalization after surgery (Cohort 2)

**eTable 4.** Known-group validity: Item scores by surgical approaches and lung disease types (Cohort 2)

**eTable 5.** Known-group validity: Item scores by chest tubes and postoperative complications (Cohort 2)

**eTable 6.** Responsiveness: changes between baseline and the day of discharge (Cohort 2)

**eTable 7.** Patient compliances from baseline to the end of the first year after discharge (Cohort 3)

**eTable 8.** Missing of ePSA-Lung items during hospitalization (Cohort 3)

This supplementary material has been provided by the authors to give readers additional information about their work.

**eMethods**

**Cohort 1: Item generation**

Initially, we invited patients with primary lung cancer who had undergone surgery to participate in qualitative interviews regarding their disease and perioperative experiences (including symptoms, psychological impact, and physical and role functioning). Purposive sampling was undertaken to select patients with various demographic and disease characteristics and treatment experiences. Each interview was conducted per semi-structured interview guidelines,^1^ lasted 20–30 mins, audio-recorded, and transcribed for further analysis. We grouped common experiences (i.e., those described by more than 20% of patients) into a candidate item pool.

Next, to investigate the clinical relevance of each item in the pool, we conducted two rounds of evaluations with an expert panel that included thoracic surgeons, nurses, and researchers. Panelists rated each item’s relevance from 0 (not relevant) to 10 (very relevant). An item with a mean rating of ≥5 and a score fluctuation within 2 points across two rounds was retained in the provisional version of the electronic Perioperative Symptom Assessment with Lung surgery (ePSA-Lung).

**Cohort 2: Item finalization**

We selected items following the five detailed analysis steps outlined below:

Step 1: First, items were selected based on the item-total score correlation, which represents the correlation of a single item with the sum of all other items in classical test theory. For the item-total score correlation, a score of 0.40 or higher was considered appropriate independence (Spearman correlation coefficient).^2^

Step 2: Exploratory factor analysis was conducted for item selection. Kaiser-Meyer-Olkin (KMO) test and Bartlett’s sphericity test values were examined to verify the application of exploratory factor analysis. A KMO value of 0.7 or higher and a p-value <0.001 from Bartlett’s sphericity test indicate the appropriateness of the data correlation analysis. The maximum likelihood method was used for exploratory factor analysis by principal component analysis. For the “representation” character quality, where each sub-factor is judged to be independent, varimax rotation, which is a direct rotation method, was applied. The final items were chosen for factor selection by checking whether there were any items with a factor loading value >0.4 across several factors.^3^

Step 3: Critical ratio (CR) method demonstrated the items’ sensitivity. The total score on the scale ranged from high to low, with the scores in the top 27% and the bottom 27% being the high- and low-score groups, respectively.^4^ The CR value was obtained using a t-test to compare the differences between the high- and low-scoring groups.

Step 4: A homogeneity test (deleted Cronbach’s α) for internal consistency was conducted. Cronbach’s α was calculated to confirm the internal consistency of the total scale. The Cronbach’s α after deleting an item is smaller than the Cronbach’s α of the total scale before deleting it.^5^

Step 5: Finally, we considered the floor and ceiling effects per item.^2,3^ The response distribution of each item was reviewed, and items with no responses of “not present (=0)” and “as bad as you can image (=10)” were also removed because those items did not convey meaningful information about the participants. Additionally, with regard to ceiling or floor effects, the percentages of patients that reached a maximum or minimum score were presented. We selected items that do not exceed 30% of ceiling or floor effects.

**Cohort 2: Reliability and validity**

Test-retest reliability, assessing the stability of the assessment tool, was conducted during the fourth week after discharge. This period was chosen as patients were reported to be in a stable state of symptoms within a 24-hour interval. During the fourth week after discharge as the stable period^6^, we selected the 21st and 22nd days (n=311, in cohort 3), given the highest patient compliance.

**Cohort 3: Clinical applicability**

We use three parameters to evaluate the clinical applicability of ePSA-Lung, including item missing, patient compliance, and patient dropout. Item missing refers to any missingness of individual items in ePSA-Lung at each time point. The patient compliance is quantified by the ratio of the number of patients who successfully completed the ePSA-Lung by the number of patients required to complete it at pre-determined time points. Patient dropout refers to instances where the patient who completely lost to follow-up for the rest time periods.

**Reference:**

1. Wei X, Yu H, Dai W, et al. Discrepancy in the perception of symptoms among patients and healthcare providers after lung cancer surgery. *Support Care Cancer*. 2021;30(2):1169-1179.

2. Nunnally, J.C. Psychometric Theory. Encyclopedia of Behavioral Medicine. 2020

3. Pesudovs K, Burr JM, Harley C, Elliott DB. The development, assessment, and selection of questionnaires. *Optom Vis Sci.* 2007;84:663-674.

4. Lau Y, Htun TP, Lim PI, et al. Psychometric properties of the Iowa Infant Feeding Attitude Scale among a multiethnic population during pregnancy. *J Hum Lact*. 2016; 32(2):315-323.

5. Rajmil L. Health measurement scales. A practical guide to their development and use, 3rd ed. *J Epidemiol Community Health*. 2005;59:250-251.

6. Dai W, Feng W, Zhang Y, et al. Patient-reported outcome-based symptom management versus usual care after lung cancer surgery: A multicenter randomized controlled trial. *J Clin Oncol*. 2022;40(9):988-996.

| **eFigure 1. Other symptom trajectories by surgical approaches (Cohort 2)** |
| --- |
|  |
| Mean scores of each item on the electronic Perioperative Symptom Assessment with Lung surgery (ePSA-Lung), including cough, disturbed sleep, fatigue, distress, and activity limitation. *P* values for the difference between single-port VATS and multi-port VATS groups from baseline to the 4^th^ postoperative day were obtained from linear mixed effect models, adjusted for smoking status, number of chest tube, and the pathology tumor node metastasis stage. In these models, the dependent variable was the score of each item, considered as a continuous variable, and the independent variables were time (also as a continuous variable), the surgical approach groups, the interaction between time and the surgical approach groups, and previously mentioned adjusted variables. The fixed effects of all independent variables and random effects for intercept and time were estimated using the maximum likelihood estimation method. The covariance structure was specified as the first-order autoregressive. |

| **eFigure 2. Symptom trajectories** **by surgical approaches (Cohort 3)** |
| --- |
| ****   |
| Mean scores of each item on the electronic Perioperative Symptom Assessment with Lung surgery (ePSA-Lung), including pain, cough, shortness of breath, disturbed sleep, fatigue, drowsiness, distress, walking difficulty, and activity limitation. *P* values for the difference between single-port VATS and multi-port VATS groups were obtained from linear mixed effect models, adjusted for age, sex, education, smoking status, Charlson Comorbidity Index (CCI) Score, number of chest tubes, and the pathology Tumor-Node-Metastasis (pTNM) stage. Item scores were modeled from baseline to POD4 during hospitalization, and from the day of discharge to the end of the first year after discharge. In these models, the dependent variable was the score of each item, considered as a continuous variable, and the independent variables were time (also as a continuous variable), surgical approach groups, the interaction between time and surgical approach groups, and previously mentioned adjusted variables. The fixed effects of all independent variables and random effects for intercept and time were estimated using the maximum likelihood estimation. The covariance structure was specified as the first-order autoregressive. |

| **eTable 1. Requirements and methods for the development of PRO instruments** | |
| --- | --- |
| **Requirements ^a^** | **Methods** |
| Hypothesize conceptual framework and potential claims | Conduct a clinical consultation regarding the content. |
|  | Patients self-report their experiences. |
| Obtain patient input | Generate a candidate item pool from surgical patients or accompanying family members via a qualitative interview. |
|  | Analyze symptoms or physical function and the frequency of each item being mentioned. |
| Cover the most common lung surgery-related symptoms or physical functions | Select items of well-to-strong clinical relevance through two rounds of evaluations with an expert panel to reach a consensus. |
| Confirm conceptual framework and draft instrument | Select the recall period, response option, format, and mode of administration. |
| Confirm ease of comprehension, comfort, and acceptability | Conduct cognitive debriefing interviews with the patients. |
| Assess measurement properties | Validate the score's reliability, construct validity, and ability to detect changes. |
| Applicable in multiple settings, modes, and electronic platforms | Ensure scale compatibility with different settings (hospital and home care), modes (paper-and-pencil, web-based, and telephone), and multiple platforms. |
| ^a^ These requirements were determined based on the Food and Drug Administration guidelines (Page 7: Development of a PRO Instrument: An Iterative Process).  Abbreviations: PRO: Patient-Reported Outcome | |

| **eTable 2. Symptoms identified through patient interview and expert evaluation (Cohort 1)** | | | |
| --- | --- | --- | --- |
| **Symptom** | **Frequency** | **Patients involved (%)** | **Relevance scores from the expert panel** |
| Pain | 967 | 100.0 | 8.51 |
| Cough | 904 | 94.9 | 8.18 |
| Shortness of breath | 491 | 89.7 | 7.19 |
| Disturbed sleep | 412 | 87.12 | 5.28 |
| Walking difficulty | 347 | 92.3 | 6.78 |
| Lack of appetite | 310 | 79.5 | 4.68 |
| Constipation | 306 | 74.4 | 4.22 |
| Anxious | 199 | 61.5 | 5.07 |
| Fatigue | 177 | 76.9 | 5.20 |
| Dry mouth | 135 | 41.0 | 3.58 |
| Fever | 104 | 30.8 | 6.20 |
| Drowsiness | 86 | 51.3 | 4.27 |
| Night sweat | 75 | 30.8 | 3.54 |
| Hoarse | 69 | 25.6 | 5.62 |
| Dizzy | 62 | 25.6 | 3.93 |
| Distress | 61 | 38.5 | 5.28 |
| Abdominal distension | 56 | 33.3 | 4.01 |
| Wound swollen | 39 | 5.1 | NA |
| Hemoptysis | 38 | 15.4 | NA |
| Diarrhea | 35 | 7.7 | NA |
| Dyspnea | 23 | 18.0 | NA |
| Nausea | 18 | 12.8 | NA |
| Itchy throat | 17 | 23.1 | 4.36 |
| Vomiting | 12 | 7.7 | NA |
| Leak | 10 | 7.7 | NA |
| Atrial fibrillation | 10 | 5.1 | NA |
| Remembering things | 7 | 10.3 | NA |
| Abbreviations: NA, Not Available. | | | |

| **eTable 3. Item scores during hospitalization after surgery (Cohort 2)** | | | | |
| --- | --- | --- | --- | --- |
| **Draft items** | **The average score of multiple time points ^a^ (n=223, Obs=1,234 ^b^)** | **The most severe score of multiple time points ^a^ (Obs=223) ^c^** | **Score=0 ^c,d^** | **Score=10 ^c^** |
| Pain | 3.62 ± 2.73 | 6.34 ± 2.42 | 231 (18.7) | 23 (1.9) |
| Cough | 3.26 ± 2.44 | 5.31 ± 2.3 | 195 (15.8) | 15 (1.2) |
| Disturbed sleep | 3.12 ± 2.55 | 5.39 ± 2.46 | 260 (21.3) | 13 (1.1) |
| Fatigue (feeling tired/weary) | 2.98 ± 2.47 | 5.14 ± 2.48 | 270 (22.0) | 12 (1.0) |
| Walking difficulty | 2.95 ± 2.85 | 5.7 ± 3.07 | 348 (29.1) | 44 (3.7) |
| Activity limitation | 2.92 ± 2.49 | 5.31 ± 2.7 | 313 (26.0) | 27 (2.3) |
| Shortness of breath | 2.87 ± 2.54 | 5.01 ± 2.67 | 328 (26.8) | 10 (0.8) |
| Drowsiness | 2.82 ± 2.49 | 5.39 ± 2.54 | 308 (25.2) | 17 (1.4) |
| Distress | 2.41 ± 2.49 | 4.57 ± 2.84 | 421 (34.5) | 15 (1.2) |
| Anxious | 2.16 ± 2.20 | 3.94 ± 2.56 | 412 (33.7) | 5 (0.4) |
| Hoarse | 2.15 ± 2.39 | 4.16 ± 2.69 | 456 (37.1) | 17 (1.4) |
| ^a^ Data is presented as means ± standard deviations.  ^b^ Represents all completed assessments of patients during hospitalization.  ^c^ Represents the most severe condition per patient during hospitalization.  ^d^ Data is presented as n (%).  Abbreviations: Obs, Observations. | | | | |

| **eTable 4. Known-group validity: Item scores by surgical approaches and lung disease types (Cohort 2)** | | | | | | | | |
| --- | --- | --- | --- | --- | --- | --- | --- | --- |
| **ePSA-Lung items** | **Surgical approach (single VATS vs. others, 119 vs. 104)** | | | | **Lung type (primary cancer vs. benign tumor or disease, 189 vs. 34)** | | | |
|  | **Type** | **Item score ^a^** | ***P* value** | **Effect size** | **Type** | **Item Score ^a^** | ***P* value** | **Effect size** |
| Pain | Single | 5.9 ± 2.42 | **0.004** | **0.39** | Primary cancer | 6.34 ± 2.38 | 0.42 | 0.14 |
|  | Others | 6.84 ± 2.34 |  |  | Benign | 5.29 ± 2.3 |  |  |
| Cough | Single port | 5.15 ± 2.29 | 0.26 | 0.15 | Primary cancer | 4.96 ± 2.67 | 0.96 | 0.01 |
|  | Others | 5.5 ± 2.31 |  |  | Benign | 5.3 ± 2.42 |  |  |
| Shortness of breath | Single port | 4.72 ± 2.65 | 0.09 | 0.23 | Primary cancer | 5.12 ± 2.43 | 0.14 | 0.27 |
|  | Others | 5.34 ± 2.68 |  |  | Benign | 5.34 ± 2.55 |  |  |
| Disturbed sleep | Single port | 5.22 ± 2.48 | 0.27 | 0.15 | Primary cancer | 4.55 ± 2.88 | 0.13 | 0.28 |
|  | Others | 5.59 ± 2.43 |  |  | Benign | 5.52 ± 3.05 |  |  |
| Fatigue (feeling tired/weary) | Single port | 4.84 ± 2.4 | 0.055 | 0.26 | Primary cancer | 5.17 ± 2.68 | 0.19 | 0.25 |
|  | Others | 5.48 ± 2.55 |  |  | Benign | 6.35 ± 2.81 |  |  |
| Drowsiness | Single port | 5.13 ± 2.62 | 0.12 | 0.21 | Primary cancer | 5.57 ± 2.37 | 0.033 | 0.38 |
|  | Others | 5.67 ± 2.43 |  |  | Benign | 5.48 ± 2.69 |  |  |
| Distress | Single port | 4.47 ± 2.83 | 0.60 | 0.07 | Primary cancer | 6.22 ± 2.7 | **0.041** | **0.39** |
|  | Others | 4.67 ± 2.86 |  |  | Benign | 5.35 ± 2.99 |  |  |
| Walking difficulty | Single port | 5.01 ± 2.95 | **<0.001** | **0.49** | Primary cancer | 5.83 ± 2.46 | **0.040** | **0.38** |
|  | Others | 6.49 ± 3.03 |  |  | Benign | 4.74 ± 2.53 |  |  |
| Activity limitation | Single port | 5.03 ± 2.56 | 0.09 | 0.22 | Primary cancer | 7.3 ± 2.85 | **0.020** | **0.42** |
|  | Others | 5.63 ± 2.83 |  |  | Benign | 6.52 ± 2.66 |  |  |
| ^a^ Data is presented as means ± standard deviations.  Abbreviations: ePSA-Lung, electronic Perioperative Symptom Assessment with Lung surgery; VATS, Video-Assisted Thoracic Surgery. | | | | | | | | |

| **eTable 5. Known-group validity: Item scores by chest tubes and postoperative complications (Cohort 2)** | | | | | | | | |
| --- | --- | --- | --- | --- | --- | --- | --- | --- |
| **ePSA-Lung items** | **Number of chest tube**  **(≤1 vs. >1, 104 vs. 119)** | | | | **Postoperative complications**  **(yes vs. no, 200 vs. 23)** | | | |
|  | **Type** | **Item Score ^a^** | ***P* value** | **Effect size** | **Type** | **Item Score ^a^** | ***P* value** | **Effect size** |
| Pain | ≤1 | 6.06 ± 2.6 | 0.11 | 0.21 | Yes | 6.39 ± 2.37 | 0.98 | 0.01 |
|  | >1 | 6.58 ± 2.23 |  |  | No | 5.32 ± 2.31 |  |  |
| Cough | ≤1 | 5.32 ± 2.47 | 0.98 | 0.00 | Yes | 5.12 ± 2.62 | 0.58 | 0.12 |
|  | >1 | 5.31 ± 2.15 |  |  | No | 5.5 ± 2.42 |  |  |
| Shortness of breath | ≤1 | 4.56 ± 2.97 | **0.018** | **0.31** | Yes | 5.23 ± 2.49 | 0.38 | 0.2 |
|  | >1 | 5.4 ± 2.33 |  |  | No | 5.54 ± 2.47 |  |  |
| Disturbed sleep | ≤1 | 5 ± 2.47 | **0.026** | **0.30** | Yes | 4.73 ± 2.84 | 0.09 | 0.38 |
|  | >1 | 5.73 ± 2.41 |  |  | No | 5.88 ± 3.05 |  |  |
| Fatigue (feeling tired/weary) | ≤1 | 4.72 ± 2.44 | **0.019** | **0.32** | Yes | 5.49 ± 2.65 | 0.67 | 0.09 |
|  | >1 | 5.5 ± 2.47 |  |  | No | 6.03 ± 2.7 |  |  |
| Drowsiness | ≤1 | 5.09 ± 2.57 | 0.10 | 0.22 | Yes | 5.29 ± 2.3 | 0.38 | 0.19 |
|  | >1 | 5.65 ± 2.5 |  |  | No | 4.38 ± 2.92 |  |  |
| Distress | ≤1 | 4.22 ± 2.89 | 0.09 | 0.23 | Yes | 4.79 ± 2.6 | 0.76 | 0.07 |
|  | >1 | 4.87 ± 2.78 |  |  | No | 4.62 ± 2.42 |  |  |
| Walking difficulty | ≤1 | 5.32 ± 3.01 | 0.08 | 0.23 | Yes | 4.53 ± 2.78 | **0.008** | **0.59** |
|  | >1 | 6.03 ± 3.1 |  |  | No | 3.65 ± 2.7 |  |  |
| Activity limitation | ≤1 | 5.13 ± 2.72 | 0.37 | 0.12 | Yes | 4.71 ± 3.04 | **0.023** | **0.51** |
|  | >1 | 5.46 ± 2.69 |  |  | No | 4.32 ± 2.81 |  |  |
| ^a^ Data is presented as means ± SDs.  Abbreviations: ePSA-Lung, electronic Perioperative Symptom Assessment with Lung surgery; SD, Standard Deviation. | | | | | | | | |

| **eTable 6. Responsiveness: changes between baseline and the day of discharge (Cohort 2)** | | | | | | | | |
| --- | --- | --- | --- | --- | --- | --- | --- | --- |
| **ePSA-Lung items** | **Timepoint** | **N** | **Item score (mean)** | **Item score (SD)** | **LCL** | **UCL** | ***P* value** | **Cohen’s *d* Effect size** |
| Pain | Discharge | 211 | 3.65 | 2.29 | 3.34 | 3.96 | **<0.001** | 1.80 |
|  | Baseline | 211 | 0.36 | 1.19 | 0.19 | 0.52 |  |  |
| Cough | Discharge | 211 | 3.61 | 2.24 | 3.31 | 3.92 | **<0.001** | 1.58 |
|  | Baseline | 211 | 0.77 | 1.22 | 0.60 | 0.93 |  |  |
| Shortness of breath | Discharge | 207 | 3.18 | 2.26 | 2.87 | 3.49 | **<0.001** | 1.50 |
|  | Baseline | 211 | 0.49 | 1.14 | 0.33 | 0.64 |  |  |
| Disturbed sleep | Discharge | 207 | 2.06 | 2.26 | 1.75 | 2.37 | **<0.001** | 1.01 |
|  | Baseline | 211 | 0.32 | 0.92 | 0.19 | 0.44 |  |  |
| Fatigue (feeling tired/weary) | Discharge | 209 | 2.79 | 2.36 | 2.47 | 3.12 | **<0.001** | 0.34 |
|  | Baseline | 211 | 2.00 | 2.33 | 1.68 | 2.31 |  |  |
| Drowsiness | Discharge | 209 | 2.88 | 2.21 | 2.57 | 3.18 | **<0.001** | 0.92 |
|  | Baseline | 211 | 1.09 | 1.66 | 0.86 | 1.31 |  |  |
| Distress | Discharge | 209 | 2.26 | 1.96 | 2.00 | 2.53 | **<0.001** | 0.75 |
|  | Baseline | 210 | 0.95 | 1.48 | 0.75 | 1.15 |  |  |
| Walking difficulty | Discharge | 210 | 2.03 | 2.01 | 1.76 | 2.30 | **0.040** | 0.20 |
|  | Baseline | 210 | 1.62 | 2.06 | 1.34 | 1.90 |  |  |
| Activity limitation | Discharge | 210 | 2.78 | 2.21 | 2.48 | 3.08 | **<0.001** | 1.24 |
|  | Baseline | 208 | 0.58 | 1.20 | 0.41 | 0.74 |  |  |
| Abbreviations: LCL, Lower Confidence Interval; ePSA-Lung, electronic Perioperative Symptom Assessment with Lung surgery; SD, Standard Deviation; UCL, Upper Confidence Interval. | | | | | | | | |

| **eTable 7. Percentage of patients who withdrew from baseline to the end of the first year after discharge** | | | | | |
| --- | --- | --- | --- | --- | --- |
| **Time point** | **Dropouts^a^** | | | **Compliance^b^** | |
|  | **Patients required to complete** | **Cumulative dropouts** | **Cumulative dropouts percentage (%)** | **Patients who completed** | **Compliance Percentage (%)** |
| Baseline | 775 | 1 | 0.13 | 766 | 98.97 |
| POD 1 | 774 | 3 | 0.39 | 687 | 88.76 |
| POD 2 | 774 | 6 | 0.77 | 658 | 85.01 |
| POD 3 | 727 | 7 | 0.90 | 568 | 78.13 |
| POD 4 | 524 | 10 | 1.29 | 328 | 62.62 |
| Discharge 0 | 775 | 14 | 1.81 | 521 | 68.46 |
| Discharge 1 | 775 | 16 | 2.06 | 602 | 79.11 |
| Discharge 2 | 775 | 18 | 2.32 | 620 | 81.47 |
| Discharge 3 | 775 | 21 | 2.71 | 629 | 82.65 |
| Discharge 4 | 775 | 22 | 2.84 | 634 | 83.31 |
| Discharge 5 | 775 | 23 | 2.97 | 637 | 83.71 |
| Discharge 6 | 775 | 23 | 2.97 | 637 | 83.71 |
| Discharge 7 | 775 | 24 | 3.10 | 627 | 82.39 |
| Discharge 8 | 775 | 27 | 3.48 | 645 | 84.76 |
| Discharge 9 | 775 | 29 | 3.74 | 618 | 81.21 |
| Discharge 10 | 775 | 29 | 3.74 | 616 | 80.95 |
| Discharge 11 | 775 | 29 | 3.74 | 614 | 80.68 |
| Discharge 12 | 775 | 31 | 4.00 | 612 | 80.42 |
| Discharge 13 | 775 | 31 | 4.00 | 606 | 79.63 |
| Discharge 14 | 775 | 31 | 4.00 | 606 | 79.63 |
| Discharge 15 | 775 | 32 | 4.13 | 595 | 78.19 |
| Discharge 16 | 775 | 34 | 4.39 | 596 | 78.32 |
| Discharge 17 | 775 | 34 | 4.39 | 586 | 77 |
| Discharge 18 | 775 | 34 | 4.39 | 580 | 76.22 |
| Discharge 19 | 775 | 35 | 4.52 | 604 | 79.37 |
| Discharge 20 | 775 | 35 | 4.52 | 585 | 76.87 |
| Discharge 21 | 775 | 36 | 4.65 | 588 | 77.27 |
| Discharge 22 | 775 | 39 | 5.03 | 594 | 78.06 |
| Discharge 23 | 775 | 40 | 5.16 | 571 | 75.03 |
| Discharge 24 | 775 | 41 | 5.29 | 569 | 74.77 |
| Discharge 25 | 775 | 43 | 5.55 | 568 | 74.64 |
| Discharge 26 | 775 | 44 | 5.68 | 557 | 73.19 |
| Discharge 27 | 775 | 46 | 5.94 | 548 | 72.01 |
| Discharge 28 | 775 | 48 | 6.19 | 541 | 71.09 |
| Discharge 29 | 775 | 48 | 6.19 | 534 | 70.17 |
| Discharge 30 | 775 | 51 | 6.58 | 524 | 68.86 |
| Discharge 37 | 775 | 54 | 6.97 | 623 | 81.87 |
| Discharge 44 | 775 | 63 | 8.13 | 611 | 80.29 |
| Discharge 51 | 775 | 71 | 9.16 | 581 | 76.35 |
| Discharge 58 | 775 | 72 | 9.29 | 591 | 77.66 |
| Discharge 65 | 775 | 78 | 10.06 | 584 | 76.74 |
| Discharge 72 | 775 | 85 | 10.97 | 587 | 77.14 |
| Discharge 79 | 775 | 90 | 11.61 | 576 | 75.69 |
| Discharge 86 | 775 | 95 | 12.26 | 578 | 75.95 |
| Discharge 120 | 775 | 112 | 14.45 | 581 | 76.35 |
| Discharge 150 | 775 | 127 | 16.39 | 578 | 75.95 |
| Discharge 180 | 775 | 137 | 17.68 | 562 | 73.85 |
| Discharge 210 | 775 | 152 | 19.61 | 562 | 73.85 |
| Discharge 240 | 775 | 167 | 21.55 | 559 | 73.46 |
| Discharge 270 | 775 | 185 | 23.87 | 543 | 71.35 |
| Discharge 300 | 775 | 206 | 26.58 | 481 | 63.21 |
| Discharge 330 | 775 | 233 | 30.06 | 499 | 65.57 |
| Discharge 360 | 775 | 265 | 34.19 | 510 | 67.02 |
| ^a^ Patient dropout refers to instances where the patient who completely lost to follow-up for the rest time period.  ^b^ The patient compliance is quantified by the ratio of the number of patients who successfully completed the ePSA-Lung by the number of patients required to complete it at pre-determined time points.  Abbreviations: POD, Postoperative day; NA, not applicable. | | | | | |

| **eTable 8. Missing of ePSA-Lung items^a^ during hospitalization (Cohort 3)** | | | | | | |
| --- | --- | --- | --- | --- | --- | --- |
| **Draft items** | **Response assessment timepoints^b^** | | | | | |
|  | **Baseline (n=766)** | **POD1 (n=687)** | **POD2 (n=658)** | **POD3 (n=568)** | **POD4 (n=328)** | **At discharge (n=521)** |
| Pain | 1 (0.1) | 2 (0.3) | 2 (0.3) | 4 (0.7) | 1 (0.3) | 3 (0.6) |
| Cough | 3 (0.4) | 4 (0.6) | 2 (0.3) | 1 (0.2) | 2 (0.6) | 2 (0.4) |
| Shortness of breath | 3 (0.4) | 8 (1.2) | 4 (0.6) | 1 (0.2) | 4 (1.2) | 3 (0.6) |
| Disturbed sleep | 4 (0.5) | 5 (0.7) | 7 (1.1) | 8 (1.4) | 4 (1.2) | 4 (0.8) |
| Fatigue (feeling tired/weary) | 3 (0.4) | 8 (1.2) | 5 (0.8) | 9 (1.6) | 3 (0.9) | 5 (1.0) |
| Drowsiness | 3 (0.4) | 6 (0.9) | 11 (1.7) | 6 (1.1) | 2 (0.6) | 4 (0.8) |
| Distress | 4 (0.5) | 7 (1.0) | **13 (2.0) ^c^** | 9 (1.6) | 4 (1.2) | 8 (1.5) |
| Walking difficulty | 3 (0.4) | 10 (1.5) | 9 (1.4) | 4 (0.7) | 4 (1.2) | 4 (0.8) |
| Activity limitation | 2 (0.3) | 11 (1.6) | 7 (1.1) | 10 (1.8) | 1 (0.3) | 10 (1.9) |
| ^a^ Item missing refers to any missingness of individual items in ePSA-Lung at each time point.  ^b^ Data is presented as n (%);  ^c^ The highest missing rates in an item of the ePSA-Lung instrument in cohort 3; the denominator of missing rate is the remaining patients in POD2 (n=658).  Abbreviations: POD, Postoperative day; ePSA-Lung, electronic Perioperative Symptom Assessment with Lung surgery. | | | | | | |
